# Meniscus extrusion on standing weightbearing ultrasound is associated with limb alignment and posterior tibial slope in healthy knees

**DOI:** 10.1002/jeo2.70631

**Published:** 2026-01-15

**Authors:** Yunseo Linda Park, Anja M. Wackerle, Jumpei Inoue, Kohei Kamada, Romed P. Vieider, Volker Musahl, Jonathan D. Hughes

**Affiliations:** ^1^ University of Pittsburgh School of Medicine Pittsburgh PA USA; ^2^ Department of Orthopaedic Surgery UPMC Freddie Fu Sports Medicine Center Pittsburgh PA USA; ^3^ Department for Orthopaedic Sports Medicine Klinikum Rechts der Isar, Technical University of Munich Munich Germany

**Keywords:** dynamic loading, healthy knee, meniscus extrusion, posterior tibial slope, ultrasound sonography

## Abstract

**Purpose:**

The purpose of this study was to report meniscus extrusion in asymptomatic knees using ultrasound sonography (US) and evaluate the association between the extrusion and joint space width, coronal alignment, and posterior tibial slope (PTS).

**Methods:**

Patients ≥18 years with meniscus allograft transplantation with a contralateral knee that was asymptomatic, Kellgren‐Lawrence grade ≤1, without a history of pathology were included. Knee pain or acute injury requiring orthopaedic evaluation, prior knee surgery, and inflammatory arthropathies were excluded. Meniscus position was captured via US in supine, bipodal, and unipodal stances. Participants underwent radiographic imaging, and joint space width, alignment, and PTS were measured. Statistical analyses included *t*‐tests, Mann–Whitney *U* tests, Pearson correlation, Friedman and repeated ANOVA for further comparisons. Statistical significance was set at *p* < 0.05.

**Results:**

Twenty‐eight patients (mean age: 39 ± 11 years, 36% females) were included. No significant differences were found between medial and lateral extrusion across stances (all *p* > 0.05; medial 2.7 mm vs. lateral 2.7 mm in supine). Extrusion CSA of the medial meniscus was greatest in the bipodal (22.4mm^2^), followed by unipodal (21.3mm^2^), then supine (16.0mm^2^) stances (*p* < 0.01). Extrusion distance of both menisci was greatest in the unipodal (medial 3.2 mm, lateral 3.1 mm), followed by bipodal (medial 3.1 mm, lateral 3.0 mm), then supine stances (medial 2.7 mm, lateral 2.7 mm, *p* < 0.01). Age, body mass index (BMI), and joint space width (medial 7.4 ± 1.5 mm, lateral 7.3 ± 1.4 mm) were not correlated with extrusion (all *p* > 0.05). For the lateral meniscus, greater varus alignment correlated with less change in extrusion from supine to unipodal (*r* = –0.70; *p* < 0.01) and bipodal (*r* = 0.52; *p* = 0.02) stances. For the medial meniscus, PTS had a positive correlation with extrusion from supine to unipodal (*r* = 0.50; *p* = 0.02) and bipodal (*r* = 0.53; *p* = 0.01) stances.

**Conclusions:**

Meniscus extrusion varied with loading conditions and bony morphology. Varus alignment was associated with less lateral meniscus extrusion, and increased PTS with greater medial meniscus extrusion. These results establish baseline values for dynamic meniscus extrusion in healthy knees, to guide US‐based monitoring after surgery.

**Level of Evidence:**

Level IV, case series.

**Abbreviations**
BMI: body mass index
CSA: cross‐sectional area
ICC: interclass coefficient
KL: Kellgren–Lawrence
MAT: meniscus allograft transplantation
MCL: medial collateral ligament
MRI: magnetic resonance imaging
OA: osteoarthritis
PA: posterior‐anterior
PTS: posterior tibial slope
US: ultrasound sonography

## INTRODUCTION

The meniscus distributes load, absorbs shock, and enhances knee stability during weightbearing. Magnetic resonance imaging (MRI) is considered the gold standard for diagnosing meniscal pathologies and early articular cartilage loss [29]; however, it is costly, time‐consuming, and limited to static, supine imaging. The meniscus plays a crucial role in distributing load across the knee, and its radial displacement beyond the tibiofemoral compartment can indicate early joint dysfunction [2]. Meniscus extrusion is more commonly observed in the medial meniscus and increases with age and body mass index (BMI) [1]. Meniscus extrusion is often associated with underlying knee pathologies, such as meniscal tears, osteoarthritis (OA), and insufficiency of the meniscotibial ligaments [8, 20, 28]. Since meniscus extrusion is believed to be greater during loading conditions and varies based on knee flexion [25, 27], static MRI may underestimate its true extent. Ultrasound sonography (US) offers the ability to dynamically assess meniscus extrusion and has demonstrated strong agreement with MRI [7, 25].

Most of the current literature focuses on meniscal extrusion in injured knees. However, the effect of load on healthy meniscus displacement remains poorly understood, and no consensus exists regarding the physiological range of extrusion. Establishing normal reference values of meniscus extrusion can allow for comparisons with pathologic meniscal conditions and provide a foundation for evaluating postoperative meniscus function. Additionally, it is not fully understood how joint space, coronal limb alignment, and posterior tibial slope (PTS) affect meniscus extrusion. In particular, PTS is a well‐established risk factor for anterior cruciate ligament (ACL) injury, ACL graft failure, and meniscal pathology [14, 16, 22, 26]; however, the relationship between PTS and meniscal extrusion remains unclear.

The purpose of this study was to report meniscus extrusion in asymptomatic knees using US and evaluate the association between the amount of extrusion and bony morphology. The primary outcome was meniscus extrusion compared across weightbearing conditions and between medial and lateral compartments. It was hypothesised that meniscus extrusion would increase with loading (supine < bipodal < unipodal) and be greater in the medial compartment. The secondary outcome was the correlation between extrusion and joint space width, coronal plane alignment, and medial PTS. It was hypothesised that meniscus extrusion would be associated with smaller joint space widths, increased medial PTS, varus alignment for medial meniscus extrusion, and valgus alignment for lateral meniscus extrusion.

## METHODS

This study was approved by the University of Pittsburgh institutional review board (STUDY22110002). This study was a retrospective review of prospectively collected data from a larger study evaluating long‐term outcomes of meniscal allograft transplantation (MAT) [31]. Informed consent was acquired from all participants before the initiation of the study. Individuals who underwent MAT were recruited for long‐term follow‐up via US and radiographic imaging. The examined knees were the contralateral knees to those that underwent MAT. Inclusion criteria included patients with an asymptomatic contralateral knee without a history of knee surgery or symptoms such as pain, swelling, stiffness, or locking, age 18 years or older, Kellgren‐Lawrence (KL) grade 1 or greater [18], and ability to undergo US testing protocol (e.g., weight‐bearing). Exclusion criteria were knee pain or acute knee injury requiring a visit to an orthopaedic surgeon, any knee surgery, or a history of inflammatory arthropathies such as rheumatoid or psoriatic arthritis.

### Ultrasound evaluation

**Figure 1:**
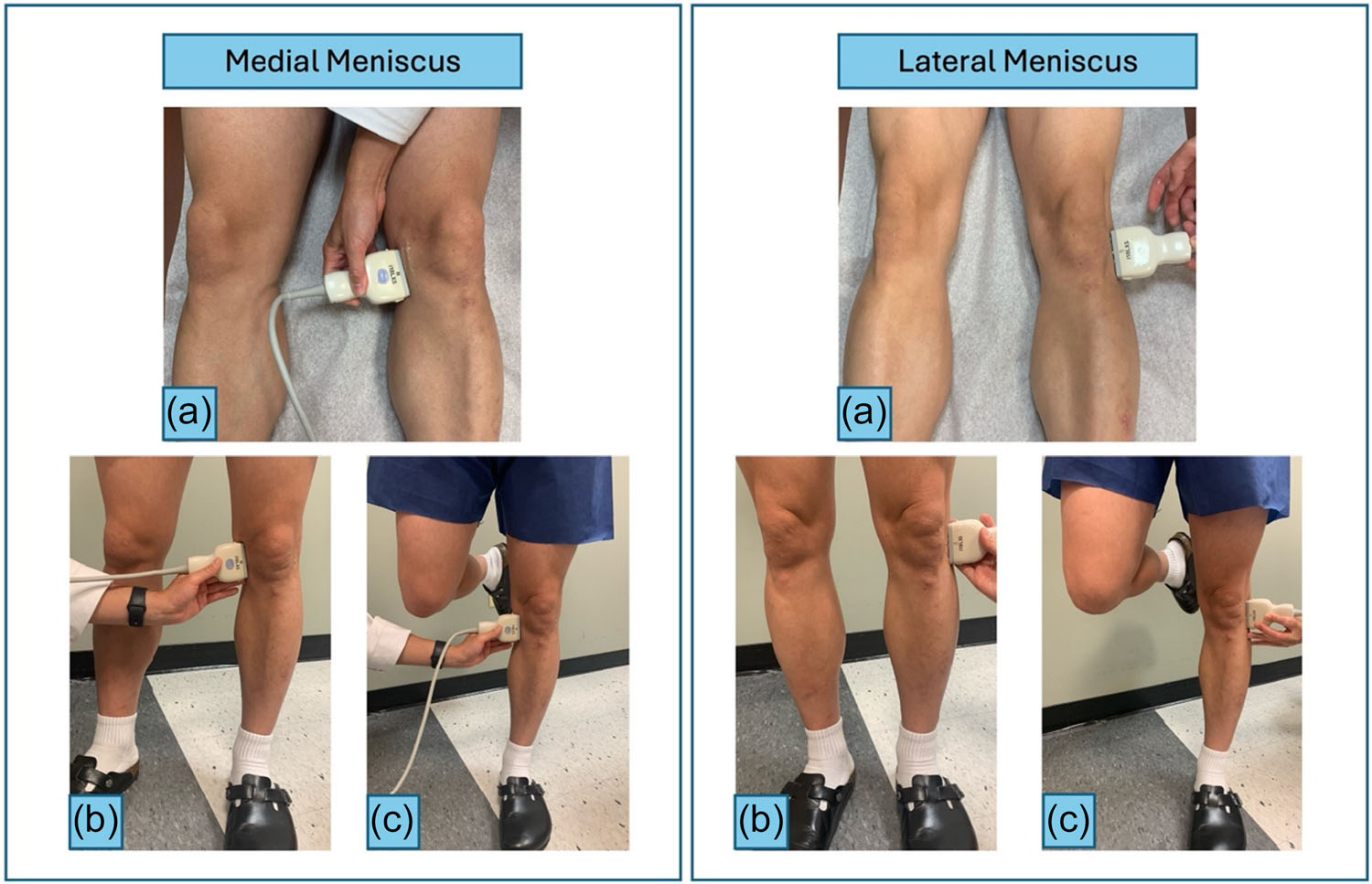
Positioning of the patient for ultrasound sonographic measurements. (a) Supine position, (b) Bipodal stance, (c) Unipodal stance. Meniscus extrusion was captured on ultrasound under these three loading conditions for both the medial and lateral meniscus.

All knees were evaluated by an experienced orthopaedic surgeon (JI) using a US system (Aplio i800; Canon Medical Systems) with a high‐frequency (18‐5 MHz) linear‐array transducer. Participants were positioned supine on the examination table with both lower extremities fully extended. The probe was placed in a vertical orientation for evaluation along the longitudinal axis. For the medial meniscus, the transducer was placed parallel to the fibre orientation of the medial collateral ligament (MCL) [17]. For the lateral meniscus, the transducer was applied just anterior to the lateral collateral ligament [30, 34]. The meniscus presented as a hypoechoic structure between the femoral and tibial cortices. The imaging depth was adjusted based on each patient. The frequency and gain were set to 18 MHz and 80 dB, respectively. Once a clear view of meniscus extrusion was obtained, images and videos were captured and saved. This process was repeated in three patient positions: supine, bipodal stance, and unipodal stance (Figure 1). For the bipodal position, participants stood upright with feet shoulder‐width apart, distributing weight equally across both limbs. For the unipodal position, they stood on the limb being evaluated while the contralateral foot was lifted off the ground to ensure a full single‐limb load.

**Figure 2:**
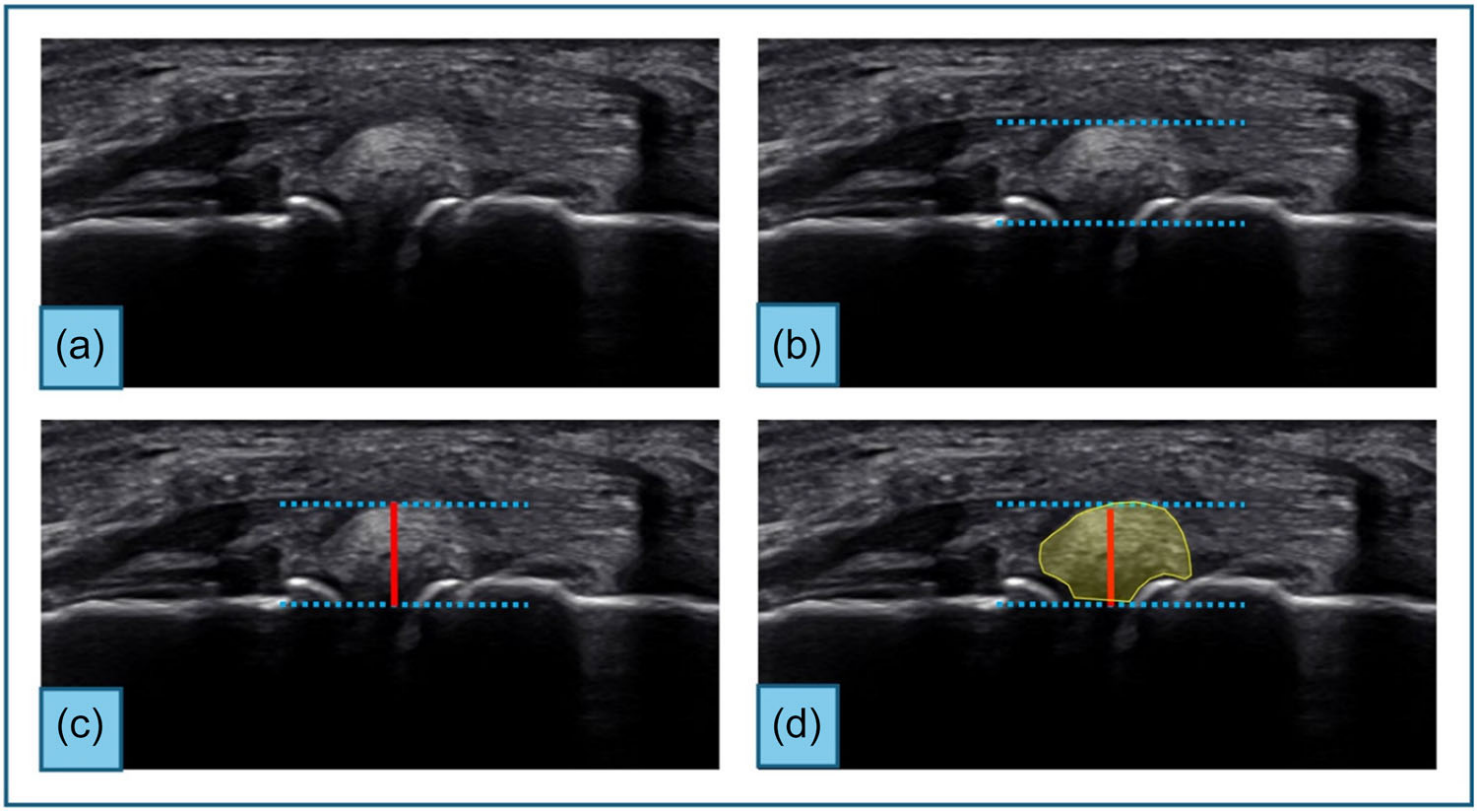
Measurement method of meniscus extrusion cross‐sectional area and distance. (a) Ultrasound image of an extruded meniscus without measurement outline. (b) Dotted blue lines are drawn tangent along femoral and tibial condyles & tangent drawn to the outermost edge of the meniscus. (c) The red line is drawn perpendicular to the dotted blue lines and represents the extrusion distance. (d) The yellow shade represents the extrusion cross‐sectional area.

Two orthopaedic surgeons independently measured the extrusion distance and extrusion cross‐sectional area (CSA) on the image acquired with US. The distance was defined as the greatest distance from a line tangent to the outermost edges of the femoral and tibial condyles to the outermost edge of the meniscus [25, 30]. Extrusion CSA was measured as the meniscus area outside of the knee joint. ImageJ software (National Institutes of Health) was used to measure the CSA in square millimetres (mm^²^) and the distance of meniscal extrusion (Figure 2).

### Radiographic evaluation

Lateral knee radiographs as well as posterior‐anterior (PA) flexion radiographs in 30 degrees of knee flexion and bipodal stance were obtained bilaterally. The following measurements were collected in both the medial and lateral compartments of the knee joint: joint space width (mm), OA grading with the KL system [18], and medial PTS. To measure the joint space of each compartment, a line tangent to both medial and lateral femoral condyles was drawn on the PA view. Two lines perpendicular to the first line were drawn at the most outer point of the tibial plateau and the medial/lateral end of the tibial spine. At the midpoint between these two landmarks, joint space width was measured as the distance between the most sclerotic lines of the femoral condyle and tibial plateau. This method was developed by our group (Supporting Information 1) [13]. Evaluation of OA features was conducted according to the KL grading system as follows: Grade 0: no radiographic features of OA, Grade 1: doubtful narrowing of joint space and possible osteophytic lipping, Grade 2: definite osteophytes and possible narrowing of joint space, Grade 3: moderate multiple osteophytes, definite narrowing of joint space, some sclerosis and possible deformity of bone ends, Grade 4: large osteophytes, marked narrowing of joint space, severe sclerosis, and definite deformity of bone ends [18]. PTS was measured as the angle between the medial tibial plateau and a line perpendicular to the tibial proximal anatomic axis, which was drawn through two diaphyseal midpoints along the tibial shaft (Supporting Information 1) [12]. Poor quality radiographs with >5 mm malalignment of the posterior femoral condyles or tibial shaft length <15 cm were excluded to screen against rotational misplacement and ensure accurate measurement of medial PTS. Patients with available previous full‐length radiographs were evaluated for coronal leg alignment by drawing a line from the centre of the femoral head to the centre of the knee and another line from the centre of the knee to the centre of the ankle. The angle formed between these two lines at the knee joint indicated the type and degree of alignment, with a deviation towards the medial side from the neutral mechanical axis being defined as varus alignment and a deviation towards the lateral side being defined as valgus alignment (Supporting Information 1). To standardise measurements on a single linear scale, valgus angles were assigned negative values, and varus angles were assigned positive values.

### Statistical methods

All statistical analyses were conducted using SPSS software version 28.0.1.1 (IBM). Descriptive statistics were reported for all demographic variables, meniscus extrusion measurements, and radiograph data. To compare extrusion between the medial and lateral menisci, normality was assessed using the Shapiro–Wilk test. Mann–Whitney *U* tests were used for variables that were not normal in either medial or lateral group, and independent samples *t* tests were used to compare normally distributed variables. To compare extrusion between different loading conditions, repeated measures ANOVA and Friedman tests were used to compare parametric and nonparametric variables, respectively. To account for differences between individuals, such as body habitus, absolute meniscal extrusion measurements were normalised to each patient's supine measurement. Higher BMI has consistently been shown to be associated with meniscus extrusion [1, 35]. The change in extrusion from supine to weight‐bearing positions was calculated for each individual, and these normalised changes were then used to evaluate correlations with age, BMI, and radiographic measurements, including joint space width, varus/valgus alignment, and medial PTS. A *p*‐value < 0.05 was considered statistically significant.

The intraclass correlation coefficient (ICC) was calculated for both intra‐ and interrater reliability. ICC values < 0.50 were considered poor, 0.50–0.75 moderate, 0.75–0.90 good, and > 0.90 excellent [21]. The intrarater ICC for the distance measurement was 0.815 (*p* < 0.01) and 0.845 (*p* < 0.01) in the lateral and medial meniscus, respectively. The intrarater ICC for CSA measurement was 0.866 (*p* < 0.01) and 0.886 (*p* < 0.01) in the lateral and medial meniscus, respectively. Measurements were performed in duplicate, with a 2‐month interval, by a single observer. To assess interrater reliability, all measurements were compared between two raters. The interrater ICC for distance measurement was 0.884 (*p* < 0.01) and 0.890 (*p* < 0.01) in the lateral and medial meniscus, respectively. The interrater ICC for CSA measurement was 0.803 (*p* < 0.01) and 0.815 (*p* < 0.01) in the lateral and medial meniscus, respectively.

A post hoc power analysis was performed using G*Power (version 3.1) to evaluate the statistical power of the primary comparison, which was medial versus lateral meniscus extrusion measured as extrusion CSA in the bipodal stance. The observed effect size was 0.43, yielding a post hoc power of 0.57, reflecting a moderate chance of detecting the observed effect. Power of secondary outcomes was also analysed for detecting the observed correlation between medial PTS and meniscus extrusion, which was one of the relationships of interest in this study. Given a sample size of 25 with available slope measurements, an observed average correlation coefficient of *r* = 0.52, and alpha = 0.05 the calculated power was 0.83, indicating good power to detect an effect of this size.

## RESULTS

Of the 44 MAT patients who were eligible for US and radiographic imaging, 28 healthy contralateral knees were available (Table 1). Sixteen patients were excluded due to diagnoses of the contralateral knee, such as knee pain treated nonoperatively, ACL injury, MCL injury, posterior cruciate ligament strain, and discoid meniscus requiring surgery. All included knees had a KL grade of 0 or 1 at the time of examination. Age and BMI did not correlate significantly with any extrusion variables (all *p* > 0.05). The extrusion CSAs and distances under different loading conditions are described in Table 2.

**Table 1:** Demographics.

	Participants (*n* = 28)
Age (years)	39 ± 11
Female sex (%)	10 (36)
BMI (kg/m^2^)	27 (24–29)
Right knee (%)	10 (36)

*Note*: Values are presented as mean ± standard deviation for normally distributed variables and median (IQR) for nonparametric variables. *n* (%) reported for categorical variables.

Abbreviations: BMI, body mass index; n, number of patients.

**Table 2:** Meniscus extrusion in millimetres, medial vs. lateral.

	Medial	Lateral	*p* value
**Supine position**			
CSA (mm^2^)	15.9 (13.5–24.8)	16.4 (13.1–23.9)	0.89
Distance (mm)	2.7 ± 0.8	2.7 ± 0.9	0.59
**Bipodal stance**			
CSA (mm^2^)	22.2 ± 7.0	19.1 ± 7.4	0.73
Distance (mm)	3.0 (2.5–3.7)	3.0 (2.4–3.5)	0.39
**Unipodal stance**			
CSA (mm^2^)	21.3 (17.5–25.2)	18.2 (14.4–23.9)	0.13
Distance (mm)	3.2 ± 0.8	3.1 ± 1.0	0.48

*Note*: Values are presented as mean ± standard deviation for normally distributed variables and median (interquartile range) for nonparametric variables.

Abbreviation: CSA, cross‐sectional area.

**Figure 3:**
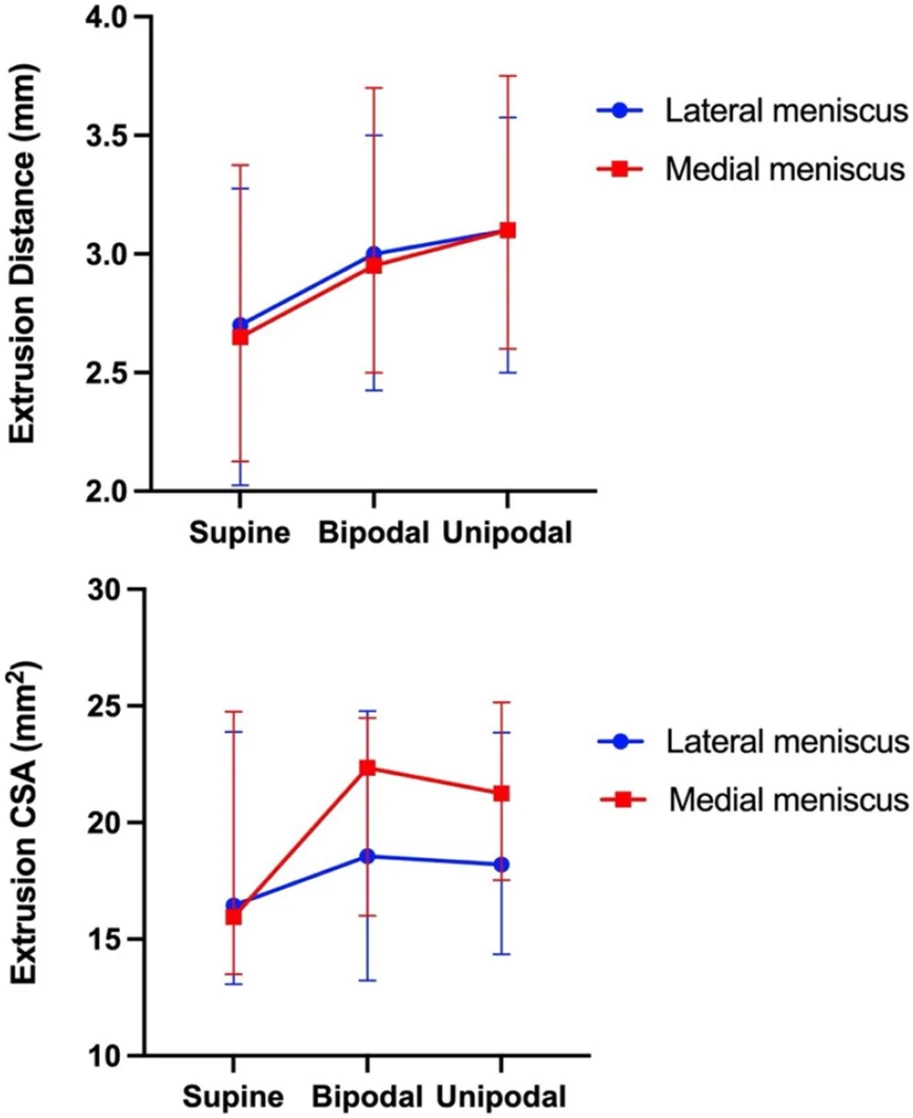
Comparison of meniscus extrusion cross‐sectional area and distance between loading conditions. Median (interquartile range) for all variables were plotted in the graphs above regardless of normality of each variable for consistency across graphs. Median extrusion distance was greatest in the unipodal stance for both medial and lateral menisci, while median extrusion cross‐sectional area was greatest in the bipodal stance for both menisci. CSA, cross‐sectional area.

When comparing extrusion between the medial and lateral meniscus, there were no statistically significant differences in all three loading conditions (all *p* > 0.05; Table 2). When comparing between loading conditions (Table 3), extrusion CSA of the medial meniscus was highest in the bipodal stance, followed by unipodal stance and supine (*p* < 0.01; Figure 3). Extrusion CSA of the lateral meniscus did not show a significant difference based on the loading condition (*p* = 0.45). Extrusion distance of both medial and lateral menisci was highest in the unipodal stance, followed by bipodal stance, and supine (*p* < 0.01; Figure 3).

**Table 3:** Meniscus extrusion in different loading conditions.

	Supine	Bipodal	Unipodal	*p* value
**Medial meniscus**				
CSA (mm^2^)	16.0 (13.5–24.8)	22.4 (16.0–24.5)	21.3 (17.5–25.2)	<0.01*
Distance (mm)	2.7 ± 0.8	3.1 ± 0.7	3.2 ± 0.8	<0.01*
**Lateral meniscus**				
CSA (mm^2^)	18.4 ± 6.9	19.1 ± 7.4	19.9 ± 6.9	0.45
Distance (mm)	2.7 (2.0–3.3)	3.0 (2.4–3.5)	3.1 (2.5–3.6)	0.01*

*Note*: Values are presented as mean ± standard deviation for normally distributed variables and median (interquartile range) for nonparametric variables. Bold values indicate statistically significant.

Abbreviation: CSA, cross‐sectional area.

When correlating change in meniscus extrusion with radiographic measurements, joint space width (medial 7.4 ± 1.5 mm; lateral 7.3 ± 1.4 mm) did not significantly correlate with any extrusion variables (all *p* > 0.05; Table 4). Twenty‐two patients had available full‐length radiographs for assessment of limb alignment. Patients demonstrated neutral (*n* = 11), varus (*n* = 7; 4.3 ± 1.8°), and valgus alignment (*n* = 4; –2.5 ± 1.0°). For the lateral meniscus, greater varus and less valgus alignment were associated with smaller changes in extrusion distance and CSA when moving from supine to unipodal (*r* = –0.70; *p* < 0.01) and bipodal (*r* = –0.52; *p* = 0.02) stances (Supporting Information 2). Medial meniscus extrusion variables did not significantly correlate with limb alignment parameters (*p* = 0.32). Measurement of medial PTS could not be obtained on three patients due to poor‐quality lateral radiographs. Overall, mean medial PTS was 11.9 ± 3.2°. For the medial meniscus, PTS positively correlated with change in extrusion distance and CSA from supine to unipodal (*r* = 0.50; *p* = 0.02) and supine to bipodal (*r* = 0.53; *p* = 0.01) stances (Supporting Information 3). Correlation between PTS and lateral meniscus extrusion was not statistically significant (*p* = 0.42).

**Table 4:** Correlation between radiographic measurements and change in meniscus extrusion from supine to weightbearing positions.

	Pearson correlation (r) with joint space	*p* value	Pearson correlation (r) with alignment a	*p* value	Pearson correlation (r) with medial PTS	*p* value
**Medial**						
Bipodal CSA (mm^2^)	−0.35	0.08	0.23	0.31	0.53	0.01*
Bipodal distance (mm)	−0.001	0.96	0.10	0.67	0.53	0.01*
Unipodal CSA (mm^2^)	0.004	0.99	0.31	0.15	0.44	0.03*
Unipodal distance (mm)	0.02	0.91	0.33	0.13	0.56	<0.01*
**Lateral**						
Bipodal CSA (mm^2^)	0.20	0.33	−0.59	<0.01*	−0.31	0.13
Bipodal distance (mm)	0.34	0.09	−0.44	0.04*	−0.15	0.47
Unipodal CSA (mm^2^)	0.35	0.08	−0.73	<0.01*	−0.19	0.36
Unipodal distance (mm)	0.28	0.17	−0.66	<0.01*	−0.07	0.73

*Note*: Bold values indicate statistically significant.

Abbreviations: CSA, cross‐sectional area; PTS, posterior tibial slope.

^a^ To standardise measurements on a single linear scale, valgus angles were assigned negative values, and varus angles were assigned positive values.

## DISCUSSION

The most important finding of this study was that meniscus extrusion did not differ significantly between the medial and lateral compartments in any loading condition. Meniscus extrusion distance in both medial and lateral compartments was greatest in the unipodal stance, while medial meniscus extrusion CSA was greatest in the bipodal stance. Additionally, valgus alignment correlated with lateral meniscus extrusion, and higher PTS correlated with medial meniscus extrusion.

Prior studies have demonstrated greater prevalence and severity of meniscus extrusion in the medial compartment, particularly in the presence of meniscus root tears [19, 32]. However, the current study, which focused on knees without meniscal pathology, did not detect a significant difference in meniscus extrusion between compartments. By comparing medial and lateral extrusion within the same knee, this study provides reliable baseline meniscus patterns.

The literature on the pattern of meniscus extrusion with altered loading conditions is mixed. A study using both MRI and US to evaluate lateral meniscus extrusion in 10 healthy knees and 17 knees with MAT found no significant difference between supine, bipodal, and unipodal stances in either group [30]. Recent US studies in healthy knees reported lateral meniscus extrusion distance decreased from supine to unipodal stance [23], while the medial meniscus extrusion distance increased in both bipodal and unipodal stances [1, 15, 23]. The present study showed that the pattern of meniscus extrusion distance differs from that of extrusion CSA. This discrepancy may be explained by the heterogeneity in the direction of meniscus extrusion, as measuring extrusion solely in the lateral or medial direction may overlook the multidirectional extrusion pattern of the meniscus. Specifically, the greater extrusion CSA of the medial meniscus in the bipodal stance may be attributed to the more extended and varus knee alignment characteristic of this posture [5, 11], which increases contact pressure across the medial compartment. Components of the lateral compartment may also contribute to this directional variability: the posterolateral capsule near the lateral collateral ligament is relatively taut and may resist extrusion, whereas the anterior corner is more lax [30]. As a result, a meniscus may exhibit substantial extrusion in terms of CSA without a corresponding increase in lateral extrusion distance. However, further studies are required to better characterise extrusion in multiple planes.

With regard to varus and valgus alignment, the Multicenter Osteoarthritis Study reported an association between varus alignment with medial meniscus extrusion on MRI with an odds ratio of 1.3 (95% CI: 1.1, 1.7) [9]. This was supported by a biomechanical study that showed varus alignment significantly increased the peak contact pressure in the medial compartment, thus increasing extrusion [33]. While the present study did not find a correlation between varus alignment and medial meniscus extrusion, it did reveal a significant relationship on the lateral side, with varus alignment being associated with reduced lateral meniscus extrusion. The absence of correlation between varus alignment and medial meniscus extrusion may reflect a cohort of near‐neutral knees, with insufficient varus magnitude to produce measurable extrusion. A larger sample size may depict a more accurate correlational relationship between variables.

The present study showed a correlation between greater medial PTS and greater medial meniscus extrusion. This suggests that increased PTS leads to not only increased tension on the ACL but also increased shear forces on the meniscus, which serves as a secondary stabiliser [3, 10]. Previous work has shown that increased PTS is associated with a higher risk of meniscus root tears due to increased compressive loads at the posterior root [4, 6, 22, 24]. Therefore, increased PTS may have biomechanical consequences beyond ACL strain, contributing to greater meniscus extrusion and potentially accelerating cartilage degeneration and OA progression.

This study has several limitations. First, all patients had a history of MAT in the contralateral knee, which may alter biomechanics and limit generalisability to truly healthy knees. Second, all US images were obtained by a single operator, which may introduce operator‐dependent bias such as variations in probe pressure and positioning. Third, sagittal‐plane US provides only one‐ and two‐dimensional measurements that may not fully capture the multidirectional nature of meniscus extrusion. Finally, the sample size was limited, resulting in low power for medial versus lateral meniscus comparisons, whereas power was higher for correlations between radiographic measurements.

## CONCLUSIONS

Meniscus extrusion varied based on loading conditions. Varus alignment was associated with less lateral meniscus extrusion, and increased medial PTS was associated with greater medial meniscus extrusion. These results establish baseline values for dynamic meniscus extrusion in healthy knees, serving as a valuable reference for US‐guided monitoring of meniscus healing after surgery.

## AUTHOR CONTRIBUTIONS

All authors contributed to the conception and design of the study. Yunseo Linda Park, Anja M. Wackerle, Jumpei Inoue and Kohei Kamada prepared the materials and collected and analysed the data. Yunseo Linda Park wrote the first draft of the manuscript, and all authors commented on earlier versions. All authors read and approved the final manuscript.

## CONFLICT OF INTEREST STATEMENT

J.D.H. has received grant support from Arthrex; education payments from Mid‐Atlantic Surgical Systems, Smith & Nephew and Medical Device Business Services; and hospitality payments from SI‐Bone. J.D.H. is on the editorial board of KSSTA. V.M. received consulting fees from Smith & Nephew and Newclip, stock or stock options from Ostesys, and is a board member of the International Society of Arthroscopy, Knee Surgery and Orthopaedic Sports Medicine (ISAKOS) and a Deputy Editor of Knee Surgery, Sports Traumatology, Arthroscopy (KSSTA). V.M. is the first vice president for the ACL Study Group. V.M. has grant funding from the NIH and DOD, but this is not pertinent to this current study. V.M. has a patent, U.S. Patent No. 9,949,684, issued on 24 April 2018.

## ETHICS STATEMENT

The methodology for this study was approved by the University of Pittsburgh Institutional Review Board (STUDY22110002). Informed consent was obtained from all individual participants included in the study.

## Supporting information

supporting information.

## Data Availability

The data that support the findings of this study are available from the corresponding author upon reasonable request.
