# Supplementary material for: Meniscus extrusion on standing weightbearing ultrasound is associated with limb alignment and posterior tibial slope in healthy knees
Source: J Exp Orthop. 2026 Jan 15;13(1):e70631. doi: 10.1002/jeo2.70631 (PMC12807492; doi:10.1002/jeo2.70631)
Supplement: Supplementary file 1 — supporting information. [file JEO2-13-e70631-s001.docx]

**Supplementary Information 1.** Measurement of posterior tibial slope, joint space width, limb alignment on radiographs


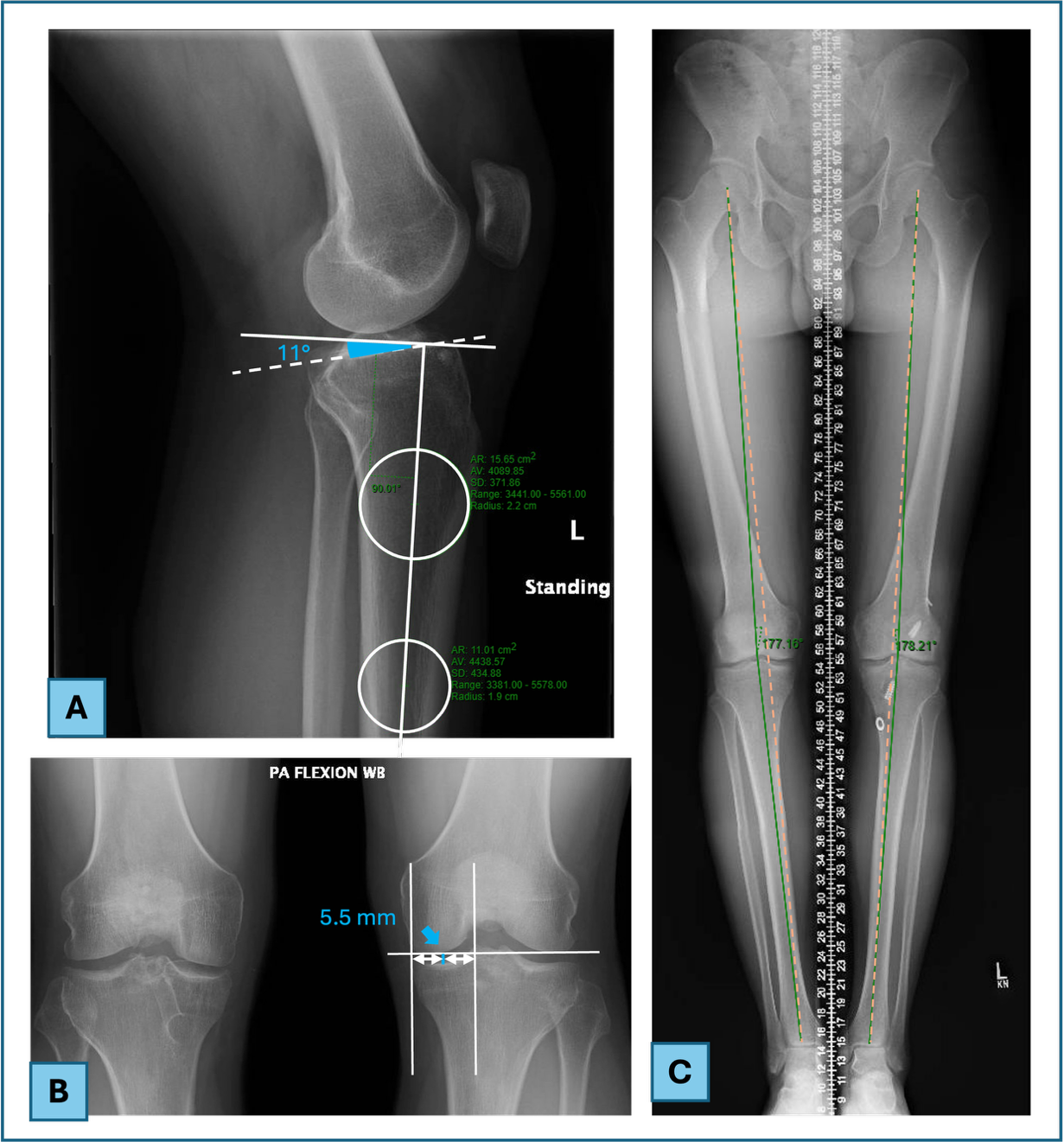


*A: Posterior tibial slope measurement on lateral view radiograph using established techniques. (Dejour et al, 1004 JBJS Br)*

*B: Joint space width measurement on posterior-anterior flexion weightbearing radiograph. Blue line indicates joint space width, measured at midpoint between outer point of tibial plateau and tibial spine. See Methods for measurement details.*

*C: Coronal leg alignment measurement on full-length radiograph. Orange dashed line indicates neutral mechanical axis. This patient is in 3° varus alignment on the right and 2° varus alignment on the left. See Methods for measurement details.*

**Supplementary Information 2.** Correlation between alignment and lateral meniscus extrusion


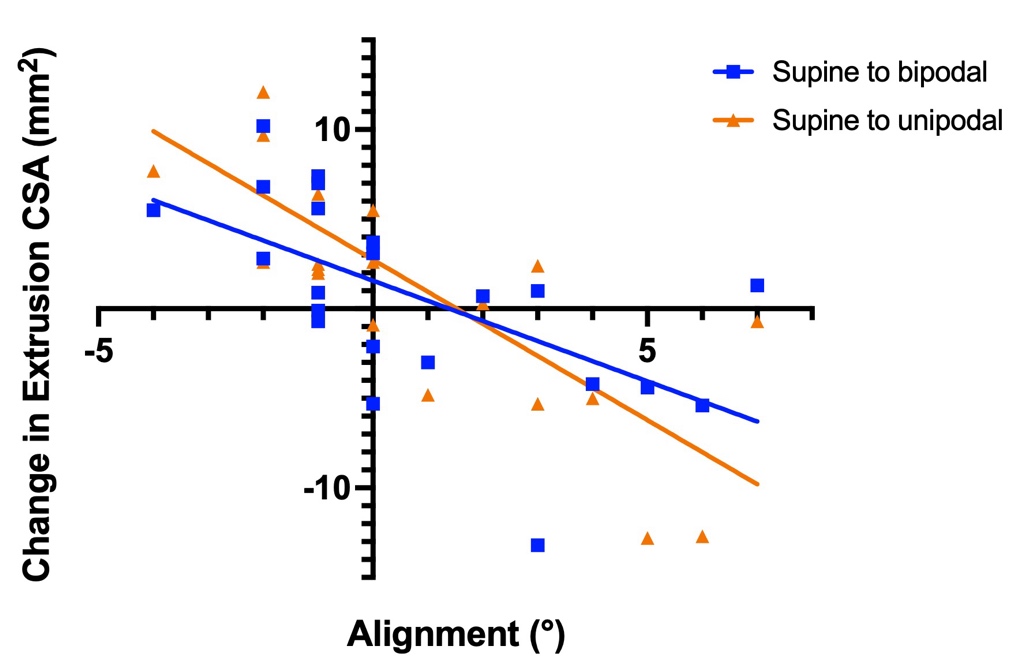


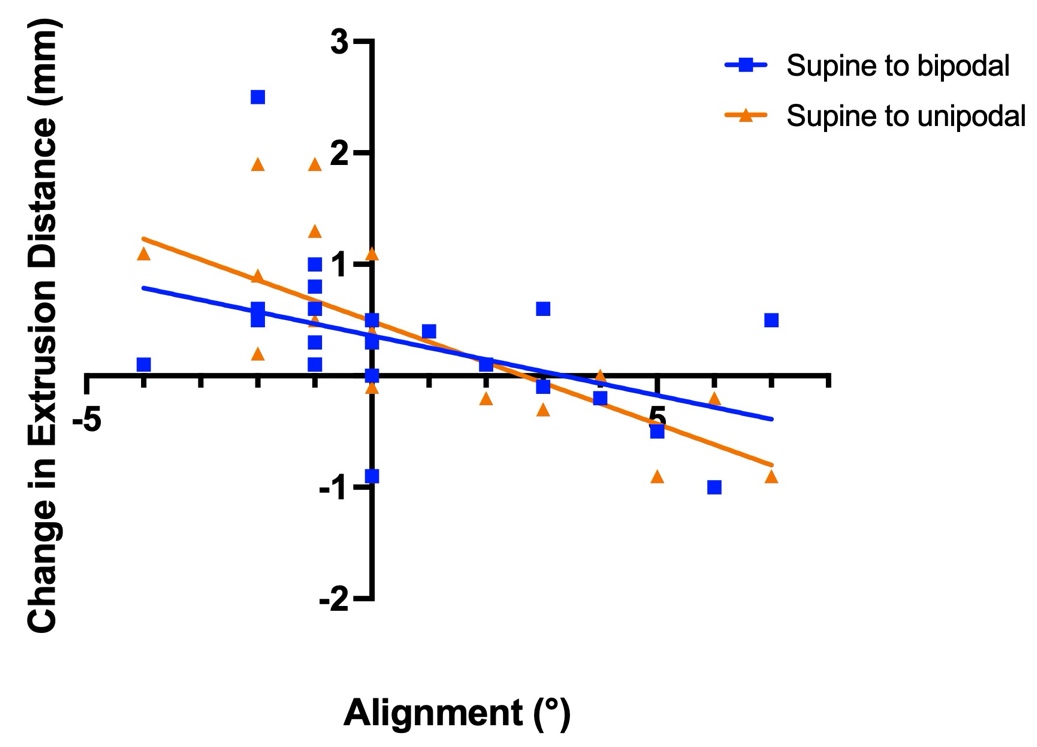


*CSA = cross-sectional area*

*To standardize measurements on a single linear scale, valgus angles were assigned negative values, and varus angles were assigned positive values.*

**Supplementary Information 3.** Correlation between medial PTS and medial meniscus extrusion
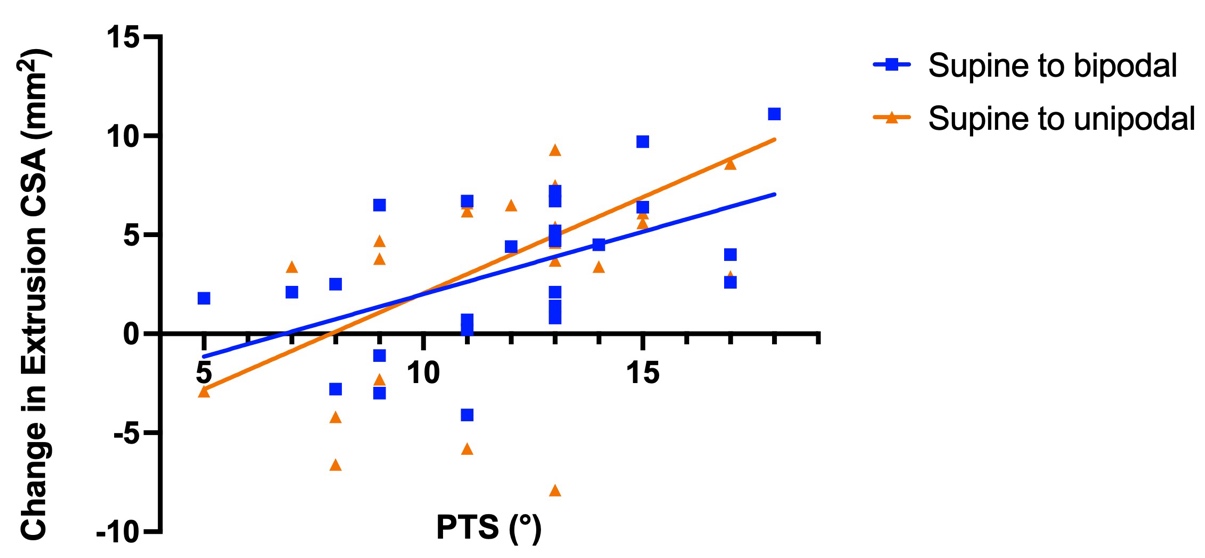


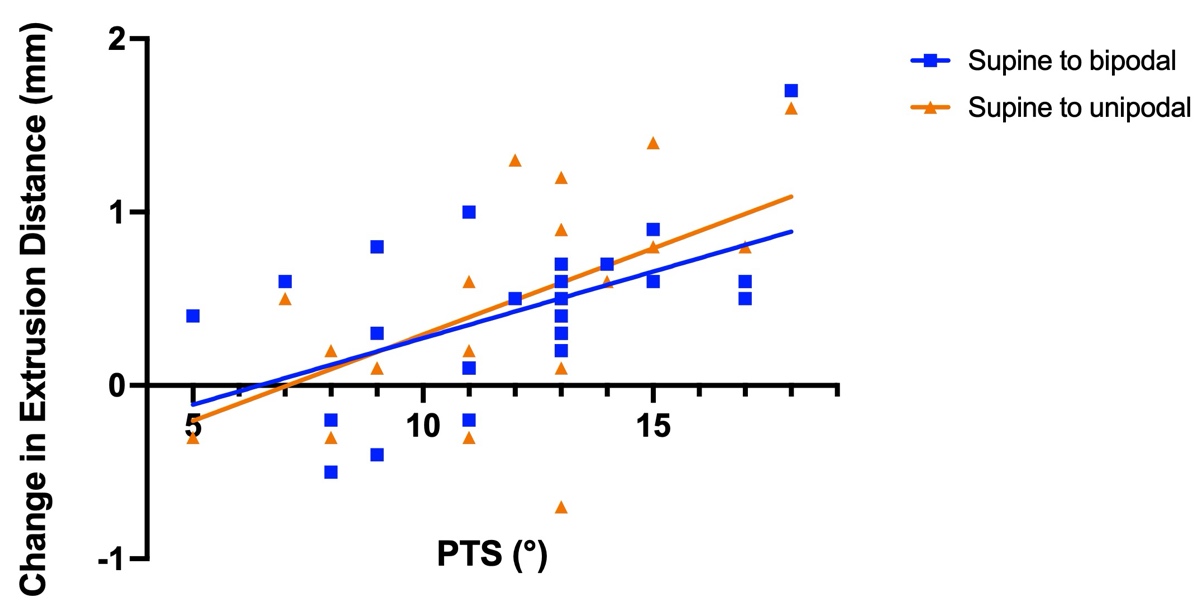


*PTS = posterior tibial slope*

**Supplementary Information 4.** Correlation between absolute meniscus extrusion and radiographic measurements

|  | **Pearson correlation (r) with joint space** | **P value** | **Pearson correlation (r) with alignment** | **P value** | **Pearson correlation (r) with PTS** | **P value** |
| --- | --- | --- | --- | --- | --- | --- |
| **Medial** |  |  |  |  |  |  |
| Supine CSA | 0.09 | 0.66 | -0.25 | 0.27 | -0.53 | **<0.01*** |
| Supine distance | 0.15 | 0.47 | -0.25 | 0.27 | -0.46 | **0.02*** |
| Bipodal CSA | -0.08 | 0.68 | -0.17 | 0.45 | -0.32 | 0.12 |
| Bipodal distance | 0.15 | 0.46 | 0.22 | 0.33 | -0.18 | 0.39 |
| Unipodal CSA | 0.08 | 0.69 | 0.03 | 0.88 | -0.14 | 0.52 |
| Unipodal distance | 0.17 | 0.40 | -0.02 | 0.92 | -0.08 | 0.71 |
| **Lateral** |  |  |  |  |  |  |
| Supine CSA | -0.18 | 0.39 | 0.22 | 0.33 | -0.46 | **0.02*** |
| Supine distance | -0.24 | 0.23 | 0.11 | 0.63 | -0.60 | **<0.01*** |
| Bipodal CSA | -0.03 | 0.90 | -0.22 | 0.33 | -0.62 | **<0.01*** |
| Bipodal distance | 0.00 | 1.00 | -0.20 | 0.38 | -0.61 | **<0.01*** |
| Unipodal CSA | 0.15 | 0.48 | -0.49 | **0.02*** | -0.62 | **<0.01*** |
| Unipodal distance | -0.03 | 0.90 | -0.29 | 0.21 | -0.63 | **<0.01*** |

*Data is not normalized to supine*
